# Hospital and Institutional News

**Published:** 1917-09-15

**Authors:** 


### HOSPITAL AND INSTITUTIONAL NEWS.

### INSTITUTIONS FOR DISCHARGED SOLDIERS.

The Ministry of Pensions has formed a Joint Institutional Committee for Scotland, which is now engaged on a comprehensive scheme for establishing in Edinburgh a hospital for discharged men suffering from neurasthenia. So far the existing institutions in Scotland have provided only for such cases among soldiers, but the time has come for the discharged man to be equally catered for. It is now reported that the estate of Craigend, Liberton, has been bought by the Corporation of Edinburgh, with a view to letting it, for this purpose, to the South-Eastern District Committee for Scotland. The promoters, indeed, hope that, when the immediate purpose has been served, the institution may become affiliated to the University as a centre for clinical study. The number of discharged soldiers in Scotland suffering from neurasthenia is believed amply to justify the new hospital. Institutions for other classes of discharged men are also being planned, including a hospital for discharged epileptics, for whom part of the Murchiston estate is now the subject of negotiations. A plan for an orthopædic hospital is also being discussed on similar lines.

### THE MEDICAL TREATMENT OF INVALIDED SOLDIERS.

We are informed that the National Health Insurance Commissioners have made new regulations establishing special arrangements for the medical treatment by general practitioners of all sailors and soldiers (including those who are uninsured) invalided from service with the Forces. These arrangements provide special facilities whereby such men can obtain the services of a doctor without delay immediately upon discharge; and they also include special provision for the treatment in sanatoria of discharged men suffering from tuberculosis.

### "THE SOLDIER'S RETURN."

The Military Hospitals Commission, Canada, has reissued a leaflet entitled "What every disabled soldier should know," which is being distributed to all invalided members of the Canadian Expeditionary Force. It sets out the facts about a man's pension or gratuity, his opportunities for training, and the arrangements made by the Provincial Governments for settling soldiers on the land, since many prefer the prospect of an outdoor life to the chances of employment in an office. Any hospital which counts Canadians among its patients should possess a store of these leaflets, which, issued from the Commission's Offices at 22 Victoria Street, Ottawa, can also be obtained, we understand, from the Commission's representatives in this country.

### A FINE PROVINCIAL SURGEON.

The presentation to Mr. W. F. Haslam, F.R.C.S., at Birmingham General Hospital was a fitting conclusion to his fifty-five years of active work. A portrait, by Mr. Edward S. Harper, which was handed over to the Lord Mayor, as one of the vice-presidents of the hospital, will now hang in the board-room, while Mr. Haslam received as a personal gift an illuminated address, inscribed by Mr. E. G. Treglown, of the School of Art, elaborately bound with gold tooling by Mr. F. Garrett. Mrs. Haslam was presented with a diamond and pearl pendant. From the fourth year of his studentship, when he gained the Cheselden Medal for surgery at the General Hospital, to the date of his appointment as the first provincial surgeon to the post of examiner under the Conjoint Examining Board, Mr. Haslam has devoted his energies to the practice of surgery at the hospital, whose reputation he has indeed helped to make in this respect.

### ITALIAN MEDALS FOR BRITISH RED CROSS WORKERS.

Mr. Geoffrey Winthrop Young, officer, and Mr. George Metcalfe and Mr. Lionel Sessions, ambulance drivers of the first British Red Cross unit for Italy, have been decorated by the King of Italy with the silver medal "For Valour," and bronze medals respectively for "repeated acts of devotion and courage in rescuing wounded under heavy fire" at the Isonzo front.

### THE LATE MR. CLARE MELHADO.

The good that a man does lives after him, at least as surely as the evil. We are happily reminded of this when we learn that a research fund connected with the Bland-Sutton Institute of Pathology has been opened, and will be associated with the name of the late Mr. F. Clare Melhado. It was he who initiated the scheme which is thus being carried forward, and it is encouraging to hear that Mr. Edmund Davis has given 1,000 guineas and Sir John and Lady Bland-Sutton £1,000 to the fund. Mr. Melhado still lives in his work, and that work continues to prosper.

### TEMPORARY APPOINTMENTS IN WAR-TIME.

As time passes, and the war drags through phases long drawn out, a succession of surprises dulls the imagination and "after the war" prospects become increasingly difficult of anticipation. We are not amongst those who fear that the cessation of hostilities will be followed by sudden chaos in medical work or otherwise. We feel, however, that the time has come to call attention to the fact that a large and increasing number of medical men and women are now holding appointments "for the duration of the war" upon somewhat indefinite conditions of tenure. It is easy and often convenient to enter into such arrangements, which lighten responsibility and give an opportunity of future reconsideration. A suggestion that a person thus temporarily appointed is, by qualification or otherwise, unsuited for permanent office is obvious and unavoidable. Such appointments are, however, frequently made temporarily, not because there is no suitable candidate, but from a desire to avoid unfairness to those, perhaps equally eligible, who may be unable to compete for them. Some risks of unfa.rness remain, for the temporary holder, if qualified for permanent appointment, not only secures a firm footing, but becomes better fitted by experience, and need have the less fear of competition when, later, he seeks election. Such an eventuality can only be avoided by the temporary appointment of someone ineligible, or with the stipulation that he shall not seek for permanent office. As matters stand at present the temporary holder may inopportunely claim to be set free, and, for that or other reasons, efforts to fill permanently the office may be made so soon after the war ceases, or at such short notice, that suitable candidates will be still tied by military or other duties. Now it is very undesirable that any haste or competition to gain early freedom from such duties should be fostered. It is therefore desirable that these temporary appointments should be made upon terms less vague than " for the duration of the war." The wisest plan would be to advertise them as " temporary," subject to termination, only, after a definite period of notice on either side. They might when reasons exist be subject to reconsideration within a certain stated period after the end of the war.

### JUBILEE OF THE METROPOLITAN ASYLUMS BOARD.

FIFTY years have elapsed since the Metropolitan Asylums Board was first established. In May 1867, when it first came into being, the Board was purely a Poor-Law organisation, having merely entrusted to it the duty of establishing asylums for the reception and relief of the sick, insane, or infirm chargeable to the Metropolitan Boards of Guardians. Since then its duties have been broadened and multiplied until now its responsibilities include the ambulance service, the training of boys, and the control of casuals—three of the widest spheres of usefulness quite distinct from the work given to the Board under the original Order. The gradual extension of the functions of the Metropolitan Asylums Board was pointed out in THE HOSPITAL of August 18, p. 394, in an article entitled "The Guardian of London's Health." The annual expenditure of the Board is now over a million and a quarter sterling, and under its control there are seventy institutions of one kind and another, with from 13,000 to 14,000 patients and other dependents. The activities of the Board now cover the whole period of life in the Metropolis from the cradle to the grave, and in these fifty years there have grown up under its widespread administration many centres of healing and relief for the sick and mentally defective that cannot be equalled in any other capital. There is little doubt that in the years to come, when war shall have ceased to worry and harass the people of London, the Metropolitan Asylums Board will be called upon to take an even more prominent place in the life and health of the Metropolis than it has done hitherto.

### HAVE THE HUT-HOSPITALS COME TO STAY?

TEMPORARY buildings, especially when used for hospital purposes, have played such a useful part during the war, and have in construction been so greatly perfected, that it is quite possible they will be widely used for a good many years to come. A writer in the *Times*, dealing with this subject, points out that we now possess many examples of semi-permanent hospitals, which in efficiency and comfort are not inferior to the big permanent institutions. The efficiency of a hospital consists not so much in the materials of construction as in the working of the organisation, the efficiency and economy of the attendance and service. Good planning, sound but simple details, good sanitation, cheerful surroundings, are all important, and these requirements can be fully met in well-designed hut-hospitals. Moreover, alterations can be carried out with the least expense, and therefore with all the more speed. Certainly, in respect of proposed additions to an old building, given sufficient ground, hut annexes seem the most sensible plan, and do not bar the way to a complete building scheme in more settled and economical times.

### LIABILITY FOR SANATORIUM TREATMENT.

AT a meeting of the Leicester Board of Guardians on the 21st ult., the case came up for consideration of a female insured consumptive aged nineteen, who was without parents and lived in lodgings with her brother, also phthisical. She was sent by the Borough M.O.H. to the Poor-Law offices, where, being an insured person, she was refused treatment. However, a temporary arrangement was made with the M.O.H. for her to be immediately admitted to the Borough Sanatorium, where the Board of Guardians as well as the Health Insurance Committee had beds. In a communication the M.O.H. pointed out that the case was one of a class which require immediate resident institutional treatment, not merely because of their tuberculosis, but on general grounds of illness and destitution; and that the difficulty in dealing with them on ordinary National Insurance lines was that selected insurance patients with tuberculosis had often to wait a considerable time before admission to a sanatorium. Under the circumstances he thought that the case ought to be dealt with by the board, but agreed with the clerk to the Guardians that it might be made a test case, to furnish a useful precedent. Representatives from the board were accordingly appointed to confer with the Sanitary Committee on the matter and report at a future meeting, the Chairman remarking that the point at issue was whether a person paying the demands of the National Insurance Act should, when sick, apply in the first instance to the Poor-Law authorities. He thought such a person should not.

### POOR LAW OR INSURANCE COMMITTEE?

THE question is one that is frequently arising, and, moreover, in circumstances where necessity must override any law, and as the workhouse hospital, or Poor-Law infirmary, or municipal hospital for advanced consumption, is much more frequently available than beds for severe pulmonary tuberculosis maintained by tuberculosis insurance committees, it is upon the former institutions that the burden of relief commonly falls. Such cases show the necessity of having available under the National Insurance Tuberculosis Authority not only sanatoria and dispensaries, but also "hospital beds" (very possibly in some sanatorium) where immediate accommodation is available in urgent need like that of the case under notice, or where, as in other instances, the patient is living in premises which can be condemned as unfit for human habitation, or where gross overcrowding exists, or where a large number of young children are exposed to tuberculosis infection from the sick person. The authorities of workhouses, etc., will always object to relieving insured persons: in short, the moral of the above affair is that every National Insurance Tuberculosis Committee should reserve some of its beds as "hospital" ones, even at the cost of delaying somewhat the admission of less severe "sanatorium" cases. The Leicester Chairman is correct in the view he takes. It is highly discreditable to those responsible for the administration of the National Insurance Acts that respectable poor persons of the non-pauper class, when attacked by tuberculosis, should be sent for treatment to the Poor-Law authorities. It is lamentable that, after six years' existence, National Insurance should thus be demonstrably proved to be a failure for want of money, of administrative thoroughness, and of a sense of responsibility and duty to the insured, who have regularly made their weekly payments and are most improperly degraded, through National Insurance, to Poor-Law agencies, from which they should be protected by those responsible for the administration of the Insurance Acts. If these brutalities continue much longer, justice may demand that somebody in responsible office shall swing for the miseries and mental torture inflicted on poor and innocent insured persons through those responsible for the administration of the National Insurance Acts. Leicester is a self-contained city, and we hope some of the citizens and the local press will make a test case, from the citizens' point of view, of the case of the poor insured woman, with which the medical officer of health dealt with such commendable consideration.

### ST. KATHARINE'S IN THE EAST.

It is impossible to glance without interest at the annual report of the new Royal College of St. Katharine, at Bromley Hall, which has been issued by the Medical Officer of Health for Poplar, Dr. F. W. Alexander. Old St. Katharine's had a curious history, and was a queer survivor of past traditions before it was removed from Regent's Park to its original district in the East End of London. The principal, Miss MacQueen, records the growth of the infant consultations, the extension of the health visiting, the training given, and the various demonstrations and classes which it is the business of the college to promote. The institution has a whole-time medical officer in Dr. Harold Waller, who takes his share in the co-operation now existing between the college and the Poplar Infant Care Association. From Bow to the Isle of Dogs the area is now receiving attention, and it is fair to record that the infantile mortality rate has fallen by 25 per cent. from the figure recorded in 1915.

### THE SWANSEA HOSPITAL'S DEBT.

Better accommodation having been provided, the waiting-list trouble at Swansea Hospital, which has been the cause of great discussion, is well on the way to being remedied. At the board's meeting last week Mr. Aeron Thomas announced that the two new small wards were nearly completed, and the laboratory—formerly used as the isolation hospital—was now being utilised as a ward. As the result of these arrangements they would be able to accommodate the male patients in the Graham Vivian Ward. There were very few female patients on the waiting list, he said, but the male surgical patients numbered ninety-one. It was reported that the balance due to the treasurer has now reached the large figure of £8,282, as compared with £5,400 last year, and Mr. Aeron Thomas paid a well-deserved tribute to the generous support of the working men, whose contributions were stated to be very satisfactory. Mr. Thomas said no one was a more ardent supporter of the Swansea Hospital than the workman. Those responsible for the management of the institution had something like £15,000 per annum to find, therefore it behoved them to take advantage of every idea to turn it to the hospital's advantage. The opinion was expressed, however, by Mr. Harry Thomas, another member of the board, that they were not tapping all the sources of revenue open to them. The only workmen who were contributing, he said, were those who did manual labour. A great many employees in local works, such as the clerks and travellers, were not asked to subscribe. He suggested that an effort should be made to obtain contributions from the whole of the employees in each and every works in the district. It was decided to make a universal appeal on the lines indicated.

### THE HOSPITAL PROBLEM IN CUMBERLAND.

We learn that one of the results of the war will be the acceptance of a larger hospital accommodation, since the waiting lists which grow at present will find some relief in the new additional buildings that have been erected in the course of the war, while building schemes checked in the interval will be no further postponed. There is also another point. Some hospitals which have been overstrained by the development of factories in their districts will probably have to serve a permanently increased population; and some of the mushroom urban areas will remain around factories that will still be busy when peace comes. An instance of this is to be found in connection with Cumberland Infirmary, where the daily average number of in-patients is now thirty-five more than in 1914. Yet it is not long since an extension was completed. Therefore a new wing is in contemplation, to meet the needs of those in the big factories of Gretna, which are expected to retain a big population even after the war. Mr. C. W. Allan Hodgson, the chairman of the committee of management, has publicly stated that as soon as the money can be found his committee proposes to add a new wing. Let us hope that these additions, here and elsewhere, will not only meet the new needs on the old scale, but be sufficient to remove the grave defect of lengthy waiting lists, which have been the chief weakness of the voluntary system, and the habit of condoning which is now losing its grip on the public.

### MR. HOULT'S BIRTHDAY GIFTS.

"Having to-day arrived at the age of seventy, in good health and in happy immediate surroundings, with a thankful heart I have decided to have the pleasure of giving away some money." In these words, Mr. Joseph Hoult, the well-known Liverpool shipowner and ex-M.P., has announced birthday gifts amounting to £70,000 in 5 per cent. War Loan Stock—or £1,000 for each year of his life—to Liverpool and Carlisle institutions. Among the gifts are £2,000 to the Stanley Hospital, Liverpool; £1,000 each to the Liverpool Royal Infirmary and the Victoria Hospital, Liscard; £1,000 to the Seamen's Orphanage, Liverpool; and £1,000 to the Holiday Home, West Kirby. The provision for Cumberland charities is due to the fact that Mr. Hoult has a residence near Penrith.

### THE NEW WARD AT MORTON BANKS.

The opening of the new ward at Keighley War Hospital, Morton Banks, is the latest step in a great and rapid development. At a cost of some £23,000 about 1,250 beds have been provided at the main hospital and its five auxiliary institutions. The new ward, which is of the latest pattern, provides for 150 patients, two-thirds of whom are accommodated within, and the remainder on the verandah. This ward has necessitated enlargements to the various departments, and those in charge were warned by Surgeon-General Bedford, Deputy-Director of Medical Services, Northern Command, that they must keep a continuous stream of wounded through the wards, and pass them on as rapidly as was possible to auxiliary hospitals, if they were to avoid a block. Even so, he believed that the operations in France would tax the hospital accommodation of the country. The administrator of the hospital is Lieut.-Colonel Scatterty, and the matron Miss Hall. The hospital, which has been well supported within and without, is now the centre of a great organisation in which voluntary help and generosity continue to play an active part.

### THE STATUS OF HORNSEY COTTAGE HOSPITAL.

A somewhat amusing discussion took place the other day on a proposal to alter the name of the Hornsey Cottage Hospital to that of the Hornsey General Hospital. It was eventually decided, after much argument, not to do so, but the local paper makes a comment on the speeches which cannot be omitted. "Some vigorous speeches," we read, "were delivered on both sides of this important question, but the arguments were so irrelevant and involved that supporters might have exchanged speeches with the opponents without affecting a single vote." A feature of the debate was a ruling of the chairman by which an amendment that the name should simply be "Hornsey Hospital" was declared out of order. Dr. Brackenbury, who was thus circumvented, had at least simplicity and directness on his side. The institution possesses twenty beds, and is therefore to be regarded as the one or the other type of hospital according to the gravity of the cases with which it is qualified to deal. That is a question of fact, which the evidence contained in the annual reports (if still published in full) can decide. A general hospital would also be expected to receive out-patients, which we believe the present hospital does not do. On the whole, then, the desire to drop the word "cottage" from the title might be most usefully proposed when the next effort is being made to improve the institution. It would offer an aim for generosity, an inducement to subscribers, and when such a fund were raised the new improvements might make the retention of the present title an obvious misnomer.

### DETECTIVES IN A MENTAL HOSPITAL.

Nothing is more irritating than a series of petty thefts, but these became so frequent at the Graylingwell Mental Hospital, Sussex, that the committee decided to consult a detective agency. In consequence of this a male and a female detective arrived at the institution, and eventually two persons, whom it was found not feasible to prosecute, were "dealt with." It would appear from this that the patients were the transgressors, and when Lieut.-Colonel H. A. Kidd in his report states that it is most difficult to eliminate the evil owing to the large number now employed, it is presumably to patients that he is referring. It is also true that a number of voluntary workers are employed in the wards, the canteen, and the workroom, but, presumably, such persons would be unlikely to feel a temptation to appropriate small sums of money. The atmosphere created by petty thefts is irritating and odious, and Lieut.-Colonel Kidd must be relieved to have discovered the offenders.

### A BEDROOM BAZAAR.

With astonishing energy, Miss Arter, a bedridden patient for seven years, has successfully organised a sale on behalf of the Royal Victoria and West Hants Hospital. The result has benefited the hospital by over £35. This is not Miss Arter's first attempt in this direction. She became an in-patient at the Boscombe branch of the hospital in April last, and while recovering from an operation enlisted the interest of her fellow-patients in a sale of work which she proposed to hold in her room. The first bazaar realised £20. The second, which was opened informally by Sylvia, Countess of Malmesbury, was personally conducted by Miss Arter from her bed in the corner of the room, which was filled with stalls displaying the work of other patients and helpers. The stalls themselves were put up by Mr. Bray, a local tradesman. The bazaar, which was opened on two successive days, contained a penny dip for the children, and has more than justified itself. In fact, a third sale of work has been decided on, and Miss Arter deserves admiration for the enthusiasm and skill with which she has carried out her own project. Mr. G. M. Saul, the secretary of the hospital, may be proud of counting these sales among the testimony of the patients to the value of the hospital and the regard in which it is held.

### CURIOUS CONDITIONAL GIFTS

The annual report of the London Hospital contains a list of perpetual donations, etc., to some of which are attached curious conditions. For instance, a recent gift, that of Mr. Charles Coleman, of War Savings Certificates to the nominal value of £100, was made on condition that a bunch of flowers, preferably forget-me-nots, to cost about one shilling, should be placed in Sophia Ward, with an inscription commemorating the death of Mrs. Coleman, whose life was prolonged by her treatment at the hospital. A gift of Stock from the Trustees of the Snowden Ward Memorial Fund is to be held and the dividends to be expended on "benefits for photographers or people in kindred callings." Other capital, provided by Mrs. Jane Keen, is to produce an income to be applied to the purchase of materials for clothing to be made by poor patients in the Light Department, "and to paying a reasonable price for such work." We notice that the expenditure of the great East End hospital is now over £150,000 a year, and that the whole of this important sum in 1916 went to defray ordinary everyday charges. The responsibility of Viscount Knutsford and the committee is very heavy, especially when one considers that the invested funds produce under £34,000, and that last year legacies contributed only the quite moderate total of £18,195. It is clear that the familiar estimate is not conservative, one penny will no longer keep the London Hospital going for a second.

### FRUIT, FISH AND VEGETABLES.

The corn harvest and fruit, which promised to be plentiful, have both suffered from the stormy weather, and much waste is likely to have occurred. Even the potato crop has suffered in many districts from disease, and in the case of fruit, the difficulty in getting a ready supply of sugar makes jam-making a difficult method of saving the supply. The hospitals have begun once more to remind the public of their claims, and of the need for gifts of fruit and vegetables to the wounded. We hope that this appeal will be successful, but we fear that no care and management can retrieve the losses incurred by the atrocious weather of the past few weeks. The Board of Agriculture and Fisheries, by the way, has issued a pamphlet on the cooking of freshwater fish, of which there is a certain supply which might well be employed. We are not aware that any steps have been taken to organise fresh-water fishing, but hospitals have so many friends that it is worth while to remind them that the killing of fresh-water fish has official sanction.

### THE TRIBUNALS AND THE HOSPITALS.

Happily there have not been many applications to the Military Tribunals for exemption from the Army for men engaged at the great hospitals, but occasionally efforts have been made to retain someone of value to the medical profession, and, as a rule, the appeal has been sympathetically heard. The governors of St. Thomas's Hospital found it necessary to ask the Lambeth Tribunal to grant exemption to a laboratory attendant, forty years of age, who was medically classified as only fit for garrison duty at home. A representative from the hospital informed the Tribunal that the applicant was in the medical school, and was engaged in teaching. An arrangement has been made by the War Office and the Board of Education whereby instructors in colleges were not to be called to the Colours, but inasmuch as the applicant was registered as a "laboratory steward" it was necessary that he should apply to the Tribunal for exemption. He was the only man left who could do this very important work. The Tribunal fully realised the seriousness of the situation, and granted the applicant six months' exemption, at the same time excusing him from service in the Volunteer Training Corps.

### NEW BREAD.

Some months have now elapsed since it was enacted that the British public were to eschew new bread, and to content itself with bread at least twelve hours old. The main idea in the restriction appears to have been the belief that most people eat more bread if it is new than when it is old, and so the amount of bread consumed would be much reduced, but at the same time there was the idea that as bread loses weight in the first few hours of its existence as a loaf, the consumer would obtain more bread for the same money. It was apparently forgotten that if the bread had to be kept for at least twelve hours it would have to be kept somewhere, and as it must not be kept in the shop, for that is equivalent to being exposed for sale, it had to be kept elsewhere, and often it was kept in living-rooms or even more insanitary places. At the same time there was a curious rule that every loaf of bread must weigh an exact number of pounds, but how this was to be attained the regulation did not state; for as bread continues to lose weight for several days, a loaf, for the greater part of its existence, cannot be of the weight required. It is now announced that there is to be some alteration in the rule relating to the keeping back of the bread for twelve hours, but what exactly is to be done is not clear. It is to be hoped that on any committee appointed to consider this matter there will be someone with a practical knowledge of bread-making.

### THIRTY YEARS OF POOR-LAW SERVICE.

After thirty years' service under the Poor Law, Mr. and Mrs. T. W. Norman have resigned their posts as master and matron of the City Road institution. Ill-health has made this inevitable, but Mr. and Mrs. Norman have clung to their work in the present circumstances as long as it was possible for them to do so, at personal sacrifice to themselves. We are glad to note that, subject to the approval of the Local Government Board, the Holborn Board of Guardians has decided to add ten years to the Poor-Law service of these officers for the purpose of calculating their superannuation allowances, out of regard for their long and devoted service. Mrs. Norman has done much good work among mothers in the maternity wards, which has been frankly acknowledged by the Government inspectors, while Mr. Norman has been president of the National Masters' and Matrons' Association which he founded. He has also acted as president of two London Poor-Law Officers' Associations.

### THE INCREASE OF PRICES.

"Since 1914 the cost of living, drugs, and petrol has advanced 100 per cent. We therefore request that our salaries be increased by the same proportion." In this manner have the three medical officers in the service of the Cricklade (Wiltshire) Board of Guardians applied for an increase of their salaries. The application does not offend by being too wordy. The facts are well marshalled, and the conclusion of the whole matter is put clearly and tersely. We shall be interested to observe the character of the Guardians' answer to the request.

### THE CRAIGLEITH QUARTERLY.

The fifth volume of the *Craigleith Hospital Chronicle* concludes with an autumn number, the first break in the regular monthly series which the shortage of paper has occasioned. At the same moment it loses the services of Miss Jane Fleming, who has edited the *Chronicle* since May 1915, and has now left to take up secretarial duties under the Y.M.C.A. at Havre. The new editor is Miss M. E. J. Cornwall, who hopes to publish the *Chronicle* quarterly, and promises its readers a Christmas number in November next. The success which attended the hospital's stall, where the work of patients was shown at a recent exhibition in favour of the auxiliary and Red Cross hospitals, has led to the organisation of a complete exhibition on the same lines for the benefit of Craigleith itself. The *Chronicle* maintains its reputation for instructive articles wherever obtainable, but the new editor invites members of the staff and the patients to be less bashful in sending contributions than of old.

### "HAPPY THOUGH WOUNDED."

As good things will, the *Gazette* of the 3rd London General Hospital, Wandsworth, has taken a new lease of life with the publication of a volume under the above title. "The Book of the Hospital" is a compilation of the best contributions of pen and pencil, which have become familiar to our readers from the comments which The Hospital has made on the successive monthly issues. It is unnecessary, therefore, to describe them again. But there will be many who, not possessing a complete set of the *Gazette*, will be glad to have in one volume a collection of the best verses, illustrations, and drawings that have made its reputation in the past. The result is 130 odd pages of handsome text, and the illustrations especially form a delightful record of hospital life in England during the war. Published at half a crown, the proceeds go to swell the Benevolent Fund of the institution, and thereby to help to maintain the supply of comforts for the wounded. What abundant proof we have from these pages of the incidents and humours of hospital life!

### THE PROBLEM OF A 31.

The August number of the magazine of the 2nd Western General Hospital, Manchester, known as the *Searchlight*, was entrusted to a new editor; but before considering the change a word may conveniently be said about his predecessor's departure. This was accompanied, in the last "Pot-pourri" for which he was responsible, by some retrospective notes, one of which is worth quotation. Writing of the early days of the institution, and the inexperience which everyone shared before the war, he says: "All the books that dealt with hospital organisation at the beginning of hostilities did not deal with numbers running into thousands, at least those which we knew. Problems had to be met and overcome; common-sense dealing only met the tremendous emergencies. One man worked the records. I well remember two solving the problem of A 31. There was no guide as to how this was to be filled in when hundreds were being admitted every day. All documents relating to discharges were compiled by one man; so were medical boards. Practically every one of those wonderful organisations, now known as departments, was worked by one man, and has grown out of that beginning. Out-patients were three or four in number at the commencement. Each man admitted had to bring in a day's rations." This scrap of history has the true air of things seen and remembered, and we are glad the ex-editor found time to insert it before he handed on his work.

### THIS WEEK'S DRUG MARKET.

The upward tendency of prices continues, and it is surprising how the demand is maintained in face of the extraordinarily high values. Olive oil is very scarce, and in face of the stoppage of exports from producing countries the scarcity is likely to become very acute; the value is about three times the pre-war figure. Other drugs which have advanced in price include ergot of rye, senega root, gum arabic, balsam of tolu, balsam of Peru, and aniseed oil. The demand for honey has improved, and prices are tending upwards. There is yet no relief of the scarcity of saccharine, and very high prices are obtainable for anything available; the new English make is not yet on the market, and the expected supplies from the United States have not yet arrived. The advanced prices of salicylic acid and sodium salicylate are not quite so firmly maintained, and supplies are not quite so scarce. Resorcin is again lower in price. Very little business has been done in quinine.

